# Effects of programmed death ligand 1 on the prognosis of neuroblastoma: A protocol for systematic review and meta analysis

**DOI:** 10.1097/MD.0000000000024920

**Published:** 2021-03-05

**Authors:** Zelin Wu, Fenggui Wei, Yawei Zou

**Affiliations:** Department of Pediatrics, The First Affiliated Hospital of Guangzhou Medical University, Guangzhou, Guangdong Province, China.

**Keywords:** meta-analysis, neuroblastoma, prognosis, programmed death ligand 1, protocol

## Abstract

**Background:**

Previous studies have investigated the prognostic role of programmed death ligand 1 (PD-L1) expression in patients with neuroblastoma, while the results are still controversial. Therefore, we conducted a meta-analysis to clarify the relationship between the expression of PD-L1 and the prognosis of neuroblastoma.

**Methods:**

Search electronic databases include PubMed, Cochrane, Embase, Scopus and Web of Science, and the search time is set to build the database until January 2021. Hazard ratio (HR) and 95% confidence interval (CI) were used to analyze the included results. Meta-analysis was performed using Stata 15.0 software.

**Results:**

This review will be disseminated in print by peer-review.

**Conclusion:**

The study will provide updated evidence for the evaluation of whether the expression of PD-L1 is associated with poor prognosis in patients with neuroblastoma.

**Ethics and dissemination:**

The private information from individuals will not be published. This systematic review also should not damage participants’ rights. Ethical approval is not available. The results may be published in a peer-reviewed journal or disseminated in relevant conferences

**OSF Registration number:**

DOI 10.17605/OSF.IO/FBCY6.

## 1 Introduction

Originated from sympathetic nervous system, neuroblastoma is the most common extracranial solid tumor in children and infants.^[1]^ It was common in abdomen (75%), followed by mediastinum (20%) and neck (5%).^[2]^ It has a high degree of malignancy and rapid progress. Meanwhile, most children are in the late stage of onset, and the prognosis of high-risk children is poor. Despite the combination of high-intensity chemotherapy, surgery, radiotherapy, and hematopoietic stem cell transplantation, the 5-year survival rate is only from 30% to 40%.^[3]^ This poor prognosis may be partly attributed to the lack of effective prognostic indicators. Therefore, it is very important and urgent to identify new and effective biomarkers related to the prognosis of neuroblastoma.

As one of the immune checkpoint molecules, programmed death ligand 1(PD-L1) is also known as B7-H1 or CD274, and its receptor programmed death 1(PD-1), namely CD279, is a type I transmembrane protein receptor.^[4]^ Under normal circumstances, PD-1/PD-L1 interaction can avoid the over-activation of immune system by inhibiting the proliferation of T cells.^[5]^ Tumor cells can inhibit T cell activation and induce tumor specific T cell apoptosis or inhibition by expressing PD-L1, which leads to tumor growth.^[6]^

At present, a number of meta-analyses suggest that the expression of PD-L1 is related to the poor prognosis of patients with many kinds of malignant tumors.^[7–11]^ However, the clinical significance of PD-L1 expression in patients with neuroblastoma is still controversial.^[12–15]^ Therefore, this study conducted a meta-analysis to analyze the relationship between the expression of PD-L1 and the clinicopathological features and prognosis of patients with neuroblastoma, and to explore its value as a marker for the prediction of curative effects.

## 2 Methods

### 2.1 Study registration

The protocol of the systematic review has been registered on Open Science Framework. The registration number is DOI 10.17605/OSF.IO/FBCY6. This meta-analysis protocol is based on the Preferred Reporting Items for Systematic Reviews and Meta-Analysis Protocols (PRISMA-P) statement guidelines.^[16]^

### 2.2 Data sources and search strategy

PubMed, Cochrane, Embase, Scopus, and Web of Science will be our electronic databases for retrieval. The retrieval time is from their inception to January 2021. The retrieval strategy will be created by all the researchers to discuss on the basis of the Cochrane handbook guidelines. The search strategy for PubMed is displayed in Table 1. The retrieval strategy can be modified according to the actual situation of other electronic databases.

**Table 1:** PubMed search strategy.

Number	Search terms
#1	Neuroblastoma[MeSH]
#2	Neuroblastomas[Title/Abstract]
#3	or/1–2
#4	Programmed death ligand 1[Title/Abstract]
#5	PD-L1[Title/Abstract]
#6	or/4–5
#7	prognos^∗^[Title/Abstract]
#8	survival[Title/Abstract]
#9	or/7–8
#10	#3 and #6 and #9

### 2.3 Inclusion criteria for study selection

The included articles must meet the following inclusion criteria:

1. All the included specimens were diagnosed as neuroblastoma based on pathology and histology and did not receive anti-PD-1/PD-L1 monoclonal antibody immunotherapy;
2. The expression of PD-L1 in related neuroblastoma tissues was detected by immunohistochemistry or tissue microarray;
3. According to the data provided, hazard ratio (HR) and 95% confidence interval (CI) can be extracted or calculated;
4. Reported PD-L1 expression and survival-related data, including overall survival (OS) and disease-free survival (DFS);
5. Patients were divided into PD-L1 positive (high) and PD-L1 negative (low);
6. The relationship between clinicopathological parameters and prognosis of PD-L1 was introduced in details.

The criteria for excluding literature are summarized as follows:

1. Relevant critical articles or conference articles and related case reports or replying emails;
2. Documents that cannot be tested in electronic databases;
3. It is impossible to accurately express PD-L1 and count the clinical characteristics and overall survival data of neuroblastoma;
4. Repeatedly published literatures.

### 2.4 Data collection and analysis

#### 2.4.1 Selection of studies

**Figure 1:**
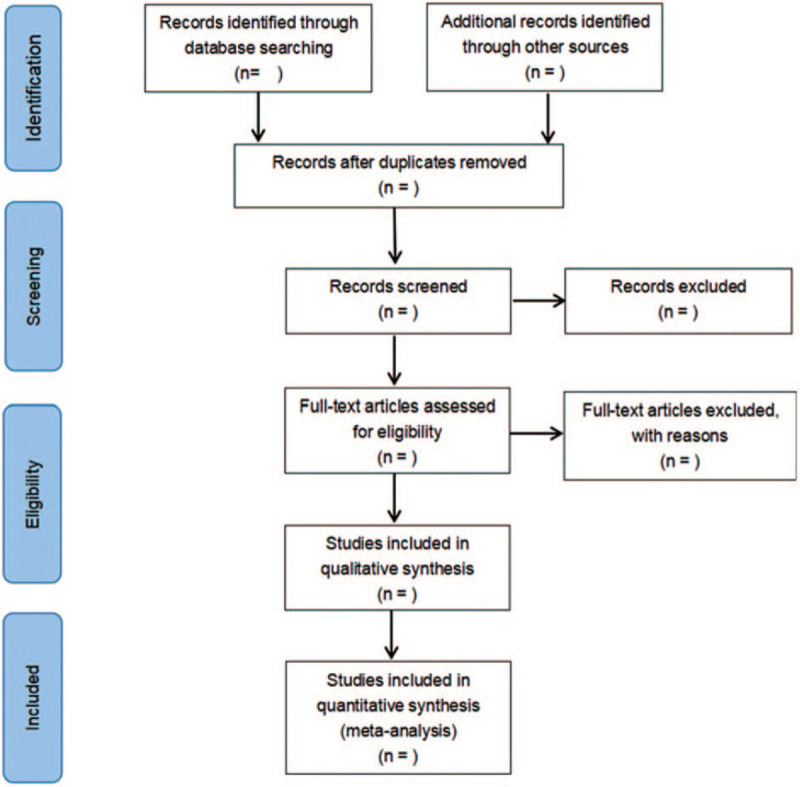
Flow diagram of literature retrieval.

Two researchers independently sift through the literature, extract data and cross-check. If they do not agree, they would seek the opinions of a third party and discuss the solution. When screening the literature, the 2 researchers first read the title and abstract. After excluding the obviously irrelevant literature, they further read the full text to determine whether it is finally included in the literature. The literature screening process is illustrated in Figure 1.

#### 2.4.2 Data extraction and management

The 2-person parallel excerpt method was adopted: Two independent researchers screened the literature according to the inclusion and exclusion criteria, and extracted the data. If there are differences, a consensus can be reached through consultation with a third researcher. Data extraction mainly includes the following information: general information (including the name, country, year and name of the first author of the literature), the number of patients, clinicopathological characteristics of patients, PD-L1 detection methods, antibodies, PD-L1 positive critical value, PD-L1 positive expression rate, OS, DFS, HR, 95%CI, *P* value, and so on. In order to minimize bias, priority was given to extract HR and 95% CI in multivariate analysis. If the data cannot be extracted directly, it can be calculated indirectly by the method introduced by Tierney et al,^[17]^ or the HR and 95% CI of the Kaplan–Meier curve can be extracted by Engauge Digitizer version 4.1 (http://digitizer.sourceforge.net/).

### 2.5 Assessment of quality in included studies

The quality of all the included studies will be evaluated by 2 reviewers independently based on the Newcastle-Ottawa scale (NOS) that is applied to evaluate the quality of observational studies.^[18]^ The NOS values arrange from 0 to 9. Studies with the score of 6 are considered to be of high quality.^[19]^

### 2.6 Measures of prognosis

OS and DFS will be taken as prognostic outcomes. The results will be expressed as HRs with 95% CIs.

### 2.7 Management of missing data

If there is insufficient or missing data in the literature, we will contact the author via email. If the data is not available, we will only analyze the available data and discuss the potential impacts.

### 2.8 Statistical analysis

Statistical analysis was performed using STATA 15.0 (STATA Corporation, College Station, TX). HR and 95% CI were utilized to evaluate the relationship between PD-L1 expression and OS and DFS. Odds ratio and 95% CIs were applied to evaluate the impacts of PD-L1 expression on clinicopathological characteristics. First, statistical heterogeneity tests were performed on the included studies. If there is no statistical heterogeneity among the included literatures (*I*^2^ < 50%, *P* ≥ .1), a fixed effect model will be used. When there exists statistical heterogeneity among the included literatures (*P* < .1, *I*^2^ > 50%), the sources of heterogeneity will be analyzed. Clinical heterogeneity will be treated by subgroup analysis. In the absence of significant clinical heterogeneity and methodological heterogeneity, statistical heterogeneity will be considered, and random effects models will be adopted for analysis. If the clinical heterogeneity of the subgroup analysis is significantly higher, no meta-analysis will be performed, with only a descriptive analysis.

### 2.9 Additional analysis

#### 2.9.1 Subgroup analysis

We will conduct a subgroup analysis based on the detection method of PD-L1 expression, race, and source of survival data.

#### 2.9.2 Sensitivity analysis

The sensitivity analysis of each index was carried out by adopting elimination method to check the stability of the results.

#### 2.9.3 Reporting bias

If the number of studies included in a certain outcome index is no less than 10, funnel chart will be used to evaluate publication bias.^[20,21]^

### 2.10 Ethics and dissemination

The content of this article does not involve moral approval or ethical review and would be presented in print or at relevant conferences.

## 3 Discussion

PD-1 and PD-L1 are a pair of negative immune costimulatory molecules.^[22–25]^ The negative immunomodulatory affects PD-L1, and signal pathway expressed on the surface of many kinds of tumor cells mediates the tumor cells that escape from the attack of immune system to some extent.^[26–28]^ Targeted blocking of PD-L1 or PD-1 shows some curative effects in clinical trials of various tumors and has a considerable prospect. Most scholars have detected the expression of PD-L1 in liver cancer, lung cancer, and other tumor tissues by immunohistochemical method. However, its expression level is different in different tumors. The expression of PD-L1 is related to the occurrence, development, treatment sensitivity, and prognosis of tumors.

Clinical trials have proved that the survival rate of children with high-risk neuroblastoma and being treated with anti-GD2 monoclonal antibody increases to 50% to 60%.^[29]^ Clinical and basic studies suggest that immunotherapy has a good application prospect in neuroblastoma. Liao et al^[12]^ used immunohistochemical staining to detect the expression of PD-L1 in 100 cases of neuroblastoma. The results revealed that the positive rate of PD-L1 was 57%. Melaiu et al^[15]^ proposed that 26 (34%) of 77 cases of neuroblastoma expressed PD-L1. Majzner et al^[30]^ proved that PD-L1 was expressed in 17 cases (14%) of neuroblastoma. The reason for different expression rates of PD-L1 in neuroblastoma may be due to the differences in the number of cases, experimental methods and scoring system of different studies, the choice of antigen repair techniques or antibodies, the number of tumor tissues in tissue samples and the reading of films by pathologists and other subjective factors. Therefore, whether the expression of PD-L1 can judge the prognosis of neuroblastoma is still controversial. In this study, meta-analysis was carried out to further explore the relationship between the expression of PD-L1 and the clinicopathological features and prognosis of patients with neuroblastoma.

Although we conducted this meta-analysis in accordance with the PRISMA guidelines strictly, and selected qualified studies with uniform criteria, there are still some limitations in this study. First, there are differences in defining the threshold for low and high PD-L1 levels, which can lead to heterogeneity. Second, all the included studies are retrospectively explored. Although we did not limit the eligible retrospective or prospective studies, the included studies were retrospective after literature selection. More prospective studies on this issue are still needed in the future. We calculated HR and 95% CI based on Kaplan–Meier survival curves, which may contain errors compared with the direct data from original studies.

In summary, this meta-analysis reveals that the overexpression of PD-L1 may be related to the poor prognosis of patients suffering from neuroblastoma. This study will provide evidence and support for PD-L1/ PD-1 immunoassay site targeted therapy for neuroblastoma.

## Author contributions

**Data curation:** Yawei Zou.

**Formal analysis:** Fenggui Wei, Yawei Zou.

**Funding acquisition:** Zelin Wu.

**Methodology:** Fenggui Wei, Yawei Zou.

**Project administration:** Zelin Wu.

**Software:** Yawei Zou.

**Supervision:** Zelin Wu.

**Validation:** Fenggui Wei and Yawei Zou.

**Visualization:** Fenggui Wei.

**Writing – original draft:** Zelin Wu.

**Writing – review & editing:** Zelin Wu.
